# Health-related quality of life in pregnant women living with HIV: a comparison of EQ-5D and SF-12

**DOI:** 10.1186/s12955-017-0731-8

**Published:** 2017-08-30

**Authors:** Xiaowen Wang, Guangping Guo, Ling Zhou, Jiarui Zheng, Xiumin Liang, Zhanqin Li, Hongzhuan Luo, Yuyan Yang, Liyuan Yang, Ting Tan, Jun Yu, Lin Lu

**Affiliations:** 1 Department of Public Health, Kunming Medical University, Yunnan, China; 2 Yunnan Centers for Disease Control and Prevention, Yunnan, China; 3 Yunnan Maternal and Child Health Care hospital, Yunnan, China; 4 Yiliang County Centers for Disease Control and Prevention, Yunnan, China; 5 Longling County Centers for Disease Control and Prevention, Yunnan, China; 6 Fengqing County Centers for Disease Control and Prevention, Yunnan, China; 7 Longchuan County Maternal and Child Health Care hospital, Yunnan, China; 8 Longling County Maternal and Child Health Care hospital, Yunnan, China; 9 Health and Family Planning Commission of Yunnan Province, No. 309, Guomao Street, Kunming, Yunnan Province China

**Keywords:** Health related quality of life, Pregnant women living with HIV, EQ-5D, SF-12, Discrimination ability, Construct validity

## Abstract

**Background:**

This paper investigates the properties and performance of the two generic measures, EQ-5D and SF-12, for Health-Related Quality of Life (HRQoL) assessments of pregnant women living with HIV in Kunming City, Yiliang County, Daguan County, Longchuan County, Tengchong County, Longling County and Fengqing County in Yunnan Province, China.

**Methods:**

As part of a screening programme for the prevention of mother-to-child transmission of HIV (PMTCT), a retrospective cross-sectional survey was conducted in the seven Maternal and Infant Health Care centers in Yunnan Province, China, between April and June of 2016. The demographic and HIV infection-related information used in the study was collected through questionnaires designed by the study’s staff. HRQoL information was collected using two generic scales: EQ-5D and SF-12.

**Results:**

A total sample of one hundred and one pregnant women with a mean age of 30.4 ± 5.1 years was investigated. Average time elapsed since infection diagnoses was 5.8 ± 3.4 years. Only one infant (1.0%) was HIV positive, and 56 (55.4%) infants were HIV negative. The HIV status of 44 (43.6%) infants was unknown. The relationship between the EQ-5D functional dimensions and the PCS-12 and the relationship between the EQ-5D anxiety/depression dimension and the MCS-12 were stronger. Those whose PCS-12 and MCS-12 scores were at the median or lower were classified as being in worse health, while those over the median were classified as being in better health. Respondents who reported no problem on each of the EQ-5D dimensions was divided according to the median SF-12 component scores. Those who scored at the median or lower than the median were classified as being in worse health, while those higher than the median were classified as being in better health. The VAS scores were also significantly different than the median split of the SF-12 scores for these subjects.

**Conclusion:**

EQ-5D and SF-12 showed a discrimination ability in measuring the HRQoL of pregnant women living with HIV. The construct validity was identified for EQ-5D and SF-12 in the study. The respective constructs of EQ-5D and EQ-VAS may not overlap. Pregnant women living with HIV in the study gave more weight to their mental health when they provided a total health rating in EQ-VAS. EQ-VAS could explain the limitations of the EQ-5D dimension scores with ceiling effects in the survey. The results of our study could help to determine the suitable HRQoL instruments for pregnant women living with HIV and provide evidence for the proper comparison of EQ-5D and SF-12.

## Background

The survival of persons with HIV has greatly improved because of the use of antiretroviral therapy (ART) globally, transforming HIV/AIDS from a terminal illness into a chronic disease [1, 2]. As other non-fatal diseases, although the mortality has been reduced and life expectancy has been extended, a rising challenge for the population living with HIV is living in full health [3]. Health-related quality of life (HRQoL) is a patient-reported outcome that features questions addressing the multidimensional nature of health [4]. HRQoL has become an important concept to understanding HIV and its chronic conditions [5].

Pregnant women living with HIV is one of the prioritized groups for HIV prevention and therapy, as is known to most of us; with programmes to prevent mother-to-child transmission, the transmission rate could be reduced to <2%, and with expanded ART, their life span could be extrapolated to the general population [6, 7]. But the information about their health status and actual living conditions is still scarce. Just as the general population living with HIV [8, 9], stigma and discrimination are still the two biggest global social phenomena that pregnant women living with HIV must face. HIV-related stigma, as an obstacle to acquiring medical services, often threatens their mental health, which is linked with poorer health outcomes. In addition, pregnant women living with HIV are a vulnerable group of individuals. A study conducted in southern India (2017) researched that the HIV-infected pregnant women experienced the intimate partner violence (IPV), which negatively influenced the mental and physical health of them. A study conducted in Brazil (2016) showed that the pregnant women living with HIV had the financial hardship because of the jobless and low income, which deteriorated their quality of life. Pregnant women living with HIV have unique roles to be both patients and mothers. Uncertainty and anxiety of foetal health status may increase the risks of mental problems. Postpartum depression (PPD) is also one of their prevalent psychological problems. A range of psychological problems, as an important part of quality of life, could have lasting effects on the child’s health [10–13].

Therefore, the HRQoL of pregnant women living with HIV warrants further study. The next step’s challenge is to choose measures that are suitable and valid for pregnant women living with HIV. The EuroQoL 5-dimensions (EQ-5D) and 12-item short-form health survey (SF-12) are two generic HRQoL measures widely used globally [14]. The SF-12 is a shortened version of the SF-36. The 12 items included in the SF-12 are a subset of those in the SF-36 and could explain at least 90% accuracy of the SF-36 [15, 16]. Applicability of the SF-12 assessment in Chinese population has been verified [17, 18]. As a generic preference-based measure of health, due to a single preference based on health status, the EQ-5D has many applications in the cost-effectiveness evaluation. The results could aid policy makers to set priorities for resource allocation [1, 19, 20].

EQ-5D and SF-12 are strongly comparable in HRQoL measurement [21]. Some researchers have tried to construct the relationship by predicting the EQ-5D utility scores from the SF-12 health survey [22]. One previous study showed that a combination may provide more coverage in measuring health [23]. Our study conducts a cross-sectional survey in a screening programme for preventing mother-to-child transmission in seven areas in Yunnan Province and simultaneously applies EQ-5D and SF-12. The objective is to investigate the properties and performance of the two generic measures of HRQoL assessments for pregnant women living with HIV. Finally, we can identify the proper instruments and provide evidence about the applicability of EQ-5D and SF-12 in measuring the HRQoL of pregnant women living with HIV.

## Methods

### Research field and subjects

As part of a screening programme for preventing mother-to-child transmission of HIV (PMTCT), we conducted a retrospective cross-sectional survey in Yunnan Province’s Maternal and Infant Health Care center and Daguan, Yiliang, Longchuan, Tengchong, Longling and Fengqing Counties’Maternal and Infant Health Care centers between April and June 2016. According to the criteria of PMTCT (if the infants are more than 18 months old, the mothers are considered to be a part of the general population living with HIV and would switch to infectious hospitals to accept regular therapy) and referring to the nearly full coverage of PMTCT services in Yunnan Province, we defined respondents as pregnant women and mothers with infants up to 18 months old. We chose the respondents using convenience sampling.

### Information and variables of interests

Demographic data was collected through questionnaires designed by the study’s staff including age, education, ethnicity, marital status, household income per year, time of HIV diagnosis, and gestational status. HRQoL information was collected using two generic questionnaires: EQ-5D (EuroQoL 5-dimensions) and SF-12 (12-item Short Form Health Survey). The survey was orally administered by a trained research team member. All the investigators received strict training before the investigation.

### Description of instruments

#### EQ-5D

The EQ-5D comprises a questionnaire and a visual analogue scale (VAS). The former is a standardized measure of health status that includes five domains about quality of daily life, such as mobility, self-care, usual activities, anxiety, and depression. Within each domain, there are three possible answers: no problem, some problems and severe problems. Through different answer combinations, a total of 243 health states are possible. The Visual Analogue Scale (VAS) assesses overall current health. The score is recorded on a vertical, visual analogue scale with the following endpoints: 0 standing for ‘worst imaginable health state’ to 100 standing for ‘best imaginable health state’ [24].

#### SF-12

The SF-12 derived from the SF-36 and measured Quality of Life with 12 items. It generates a profile of respondents’ HRQoL across eight domains: physical function (PH), role physical (RP), bodily pain (BP), general health (GH), vitality (V), social function (SF), role emotion (RE), and mental health (MH). Finally, the SF-12 generates a summary of physical functional scores (PCS) and mental functional scores (MCS). The PCS is calculated based on a combination of physical functioning, role physical, bodily pain and general health scores. The MCS is calculated based on a combination of vitality, social functioning, role emotional, and mental health scores [25].

### Statistical analysis

#### Scoring EQ-5D

Because scores of HRQoL cannot be acquired directly through the EQ-5D health state description system, we needed to use a population-based preference trade-off time (TTO) model to transform the measures into health-utility scores [26]. In the study, we used the Japanese TTO method to qualify it [27]. The Japanese value set is shown in Table 1. C is a constant. MO2, SC2, UA2, PD2 and AD2 represent the second level in mobility, self-care, usual activities, pain/discomfort and anxiety/depression, respectively. MO3, SC3, UA3, PD3 and AD3 represent the third level in mobility, self-care, usual activities, pain/discomfort and anxiety/depression, respectively. For example, the utility score for the health status “21,123” is U = 1–0.152-0.075-0-0-0.080–0.112 = 0.581. The utility score for the health status “11,111” is 1, which stands for no problem in any of the five dimensions.

**Table 1:** Japanese value set for EQ-5D health status

C	MO2	MO3	SC2	SC3	UA2	UA3	PD2	PD3	AD2	AD3
0.152	0.075	0.418	0.054	0.102	0.044	0.132	0.08	0.194	0.063	0.112

#### Scoring SF-12

The SF-12 scores are calculated using the 2nd edition of standard US instrument scoring algorithms (SF-12v2), including calculating PCS and MCS. The details can be reviewed in this professional manuscript.

#### Data analysis

Descriptive statistics show the demographic characteristics of the study sample. Because previous studies showed some associated factors that may influence the HRQoL of pregnant women living with HIV [10, 11], we include relevant variables into demographic variables. One-way ANOVA and Chi-square tests or Fisher’s exact method are conducted to compare the differences in responses of the EQ-5D health state description system, mean EQ-VAS scores, mean PCS-12 and MCS-12 scores among the different demographic characteristics.

Data analysis mainly includes three parts: the discrimination ability of the SF-12, the discrimination ability of EQ-5D health utility scores and EQ-VAS scores and an assessment of the correlations between EQ-5D and SF-12 component scores.

In analysing the discrimination ability of the SF-12 scores, the key point is whether SF-12 is sensitive enough to detect a difference between reports of “have a problem” and “no problem” in EQ-5D dimensions. First, respondents were divided into three levels in EQ-5D dimensions: no problem (code = 1), some problems (code = 2) and severe problems (code = 3). Then, we compared mean PCS-12 scores and mean MCS-12 scores across these three levels to infer the discriminating ability of the SF-12 as an HRQoL measurement of pregnant women living with HIV. We also calculated the value of η-sq.(ss_model_-ss_total_) to show the relationship, which means the strength of the relationship in an ANOVA without the influence of the sample size.

In analysing the discrimination ability of EQ-5D health utility scores and EQ-VAS scores, the key point is whether EQ-5D health utility scores and EQ-VAS scores are sensitive enough to discriminate the differences between cut-off scores of PCS-12 and MCS-12, respectively. First, respondents were divided into two groups by the mean scores of PCS-12 and MCS-12 at the median or lower (SF-12 ≤ median) and higher than mean scores (SF-12 > median). In addition, we compared mean EQ-5D health utility scores and mean EQ-VAS scores across this category to infer discrimination ability of EQ-5D health utility scores and EQ-VAS scores, respectively, as an HRQoL measurement of pregnant women living with HIV. We further assessed the discrimination ability of the SF-12 in respondents who reported having no problem on each of the EQ-5D dimensions.

We used a multitrait-multimethod (MTMM) matrix to assess the correlations between EQ-5D and SF-12 component scores by Pearson correlation analysis. MTMM matrix is a psychometric method to assess the degree that an instrument adequately reflects the dimensions of the measured construct [28], which analyses the degree of the agreement between component scores of EQ-5D and SF-12 in our study. The correlation coefficient can be defined at five levels: 1 is perfect, 0.7 to 0.9 is strong, 0.4 to 0.69 is moderate, 0.1 to 0.39 is weak and 0 is no correlation [29].

All analyses were performed using STATA 12.0 with the level of significance set at 0.05.

## Results

### Respondent sample

The study enrolled a total of one hundred and one pregnant women with a mean age of 30.4 ± 5.1 years, ranging from 20 to 42. Sixty-seven respondents (66.3%) were of the Han nationality, while others were from minority ethnic groups, including Dai, Yi, Jingpo, Lisu and Bai. Nighty-seven respondents (96.0%) declared themselves married or cohabiting. Seventy-three (72.3%) respondents reported completing less than 9 years of education. In the last 3 months, 74 (73.2%) respondents reported unemployment, taking care of their infants at home or preparing for the delivery. Sixty-six (65.3%) respondents had a household income per year exceeding 10,000 yuan. The average time since diagnosis was 5.8 ± 3.4 years. Only one infant (1.0%) was HIV positive. Fifty-six (55.4%) infants were HIV negative. The HIV status of 44 (43.6%) infants was uncertain (Table 2).

**Table 2:** Study sample characteristics

Characteristic	Distribution (%)	
Age (Years)	18–30	38(37.6)
	30–45	63(62.4)
Race/ethnicity	Han nationality	67(66.3)
	Minority ethnic group	34(33.7)
Marital status	Married/Cohabitating	97(96.0)
	Separated/divorced	4(4.0)
Education level	<9 years	73(72.3)
	≥ 9 years	28(27.7)
Employment status in recent three months	Not working	74(7.3)
	part-time	3(3.0)
	full-time	24(23.8)
Household income per year (CNY)	<10,000 yuan	11(10.9)
	10,000 to 50,000 yuan	66(65.3)
	>50,000 yuan	24(23.8)
Time since diagnosis in years	< 1 year	4(4.0)
	1to 5 years	37(36.6)
	>5 years	60(59.4)
Gestational status	pre-delivery	35(34.7)
	post-delivery	66(65.3)
Infant infection status*	HIV positive	1(1.0)
	HIV negative	56(55.4)
	Unknown	44(43.6)

*Infant must be at 18 months old could finally diagnosed with HIV negative or positive

### EQ-5D health status

**Fig. 1:**
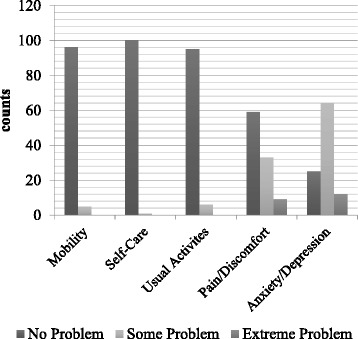
Self-rated health status-distribution

**Fig. 2:**
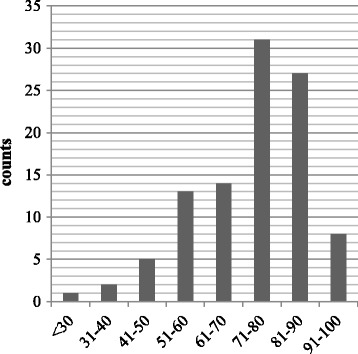
EQ-VAS scores of EQ-5D responses

The distribution of responses of the EQ-5D dimensions was skewed, with the median response being “no problem” on the first four dimensions and “some problem” on the anxiety/depression dimension (Fig. 1). The evidence showed a ceiling effect of the EQ-5D dimensions, with 56.4% of the respondents at the “ceiling” status of the functional dimensions. The ceiling effect was less apparent in the distribution of the VAS scores (Fig. 2). The mean score was 75.77 (SD = 15.17), and the median was 80.00 (IQR = 16.00).

Only a portion of the EQ-5D health utility scores was included in the study, as not all outcomes were represented in this population. There were 12 (7 + 5 “other states”) health states represented by the participants. It was found that seven health states accounted for approximately 90.0% of respondents in the survey (Table 3). Except for the five other health vector combinations that occurred among the sample, there were 231 possible health vector combinations that did not appear in this survey. On all five dimensions, 16.8% of the respondents indicated ‘no problem’.

**Table 3:** Frequency of self-reported EQ-5D health states

Vector	n	% of Total	Cumulative %
11,111	17	16.80	16.80
11,121	6	5.94	22.74
11,112	35	34.65	57.39
11,122	21	20.79	78.18
11,113	5	4.95	83.13
11,132	4	3.96	87.09
21,222	3	2.97	90.06
(5 Others)	10	9.90	99.96
Total	101	100	-

The distributions of the respondents indicating a problem on the EQ-5D by demographic and some HIV-related variables are presented in Table 3. The differences in household income per year were significant for the mobility dimension (*χ* ^2^ = 13.340, *P* = 0.001) and the usual activities dimension (*χ* ^2^ = 10.094, *P* = 0.006). The differences in education level were significant for VAS scores (*F* = 7.52, *P* = 0.007). Relationships were not observed between the demographic and HIV-related variables and EQ-5D dimensions, VAS scores for the respondents (Table 4).

**Table 4:** Responses to EQ-5D by demographic variables

Variable	*n*	% responding with a problem (moderate to extreme)										VAS mean (SD)	*P*
		Mobility	*P*	Self-care	*P*	Usual activities	*P*	Pain/discomfort	*P*	Anxiety/depression	*P*		
Age (Years)													
18–30	38	2.63	0.65	2.63	0.38	2.63	0.41	36.84	0.45	71.05	0.45	74.95(14.40)	0.56
30–45	63	6.35		0.00		7.94		44.44		77.78		76.27(15.71)	
Race/ethnicity													
Han nationality	67	7.46	0.17	1.49	1.00	7.46	0.66	38.81	0.43	77.61	0.44	75.12(15.53)	0.55
Minority ethnic group	34	0.00		0.00		2.94		47.06		70.59		77.06(14.57)	
Marital status													
Married/Cohabiting	97	5.15	1.00	1.03	1.00	6.19	1.00	41.24	1.00	75.26	1.00	75.70(15.30)	0.82
Separated/divorced	4	0.00		0.00		0.00		50.00		75.00		77.50(13.23)	
Education level													
< 9 years	73	5.48	1.00	1.37	1.00	6.85	1.00	42.47	0.77	79.45	0.11	73.29(15.33)	0.007
≧ 9 years	28	3.57		0.00		3.57		39.29		64.29		82.25(12.88)	
Employment status in recent three months													
Not working	74	6.76	-	1.35	-	8.11	0.67	43.24	0.47	74.32	0.72	74.36(15.54)	0.28
Employed part-time	3	0.00		0.00		0.00		66.67		100.00		83.33(20.82)	
Employed full-time	24	0.00		0.00		4.17		33.33		75.00		79.17(13.08)	
Household income per year													
< 10,000 yuan	11	27.27	0.001	9.09	-	27.27	0.01	45.45	0.25	81.82	0.32	73.18(10.31)	0.11
10,000 to 50,000 yuan	66	1.52		0.00		3.03		43.94		71.21		74.14(15.52)	
> 50,000 yuan	24	2.94		0.00		2.94		23.53		58.82		81.46(15.14)	
Time of diagnosis in years													
< 1 year	4	0.00	-	0.00	-	0.00	-	25.00	0.76	100.00	0.39	62.50(20.62)	0.13
1 to 5 years	37	0.00		0.00		0.00		40.54		67.57		78.19(12.47)	
> 5 years	60	8.33		1.67		0.60		43.33		78.33		75.17(16.05)	
Gestation status													
Pre-delivery	35	5.71	1.00	0.00	1.00	2.86	0.66	48.57	0.30	74.29	0.87	74.29(13.89)	0.48
Post-delivery	66	4.55		1.52		7.58		37.88		75.76		76.56(15.85)	
Infant infection status													
HIV positive/unknown	45	8.89	0.17	2.22	0.45	6.67	0.65	40.00	0.27	75.56	0.95	73.22(15.11)	0.13
HIV negative	56	1.79		0.00		3.57		42.86		75.00		77.82(15.03)	

### SF-12 composite scale scores

**Fig. 3:**
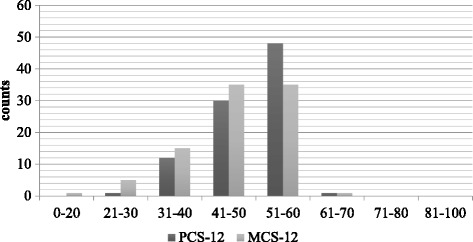
SF-12 Composite Score

The mean score of PCS-12 was 49.91 ± 7.04, and that of MCS-12 was 46.48 ± 9.12. The distributions of both component scores were somewhat skewed, with the median scores greater than the mean scores, at 51.04 and 47.23 for the PCS-12 and MCS-12, respectively (Fig. 3). The maximum scores were 63.10 and 68.02 for the PCS-12 and MCS-12, respectively.

No significant differences were observed in PCS-12 and MCS-12 scores across all demographic and HIV-related variables except PCS-12 scores in marital status (*F* = 4.32, *P* = 0.040) (Table 5).

**Table 5:** Mean SF-12 components scores by demographic variables

Variable	*n*	PCS-12		MCS-12	
		Mean(SD)	*P*	Mean(SD)	*P*
Age(Years)					
18–30	38	49.91(6.52)	0.64	46.48(7.97)	0.08
30–45	63	50.16(7.37)		45.26(9.61)	
Race/ethnicity					
Han nationality	67	50.02(7.15)	0.90	46.50(9.29)	0.64
Minority ethnic group	34	49.90(6.90)		46.40(8.90)	
Marital status					
Married/Cohabitating	97	49.62(7.02)	0.04	46.41(9.17)	0.72
Separated/divorced	4	56.95(1.89)		48.11(9.05)	
Education level					
≤ 9 years	73	49.67(7.75)	0.59	46.06(8.86)	0.45
> 9 years	28	50.52(4.78)		47.59(9.84)	
Employment status in recent three months					
Not working	74	49.77(7.08)	0.93	46.24(9.25)	0.87
part-time	3	49.38(3.93)		45.64(4.41)	
full-time	24	50.39(7.40)		47.32(9.35)	
Household income per year(CNY)					
< 10,000 yuan	11	46.87(9.42)	0.25	43.58(7.65)	0.52
10,000 to50,000 yuan	66	49.96(7.01)		47.00(9.95)	
> 50,000 yuan	24	51.16(5.63)		46.39(7.19)	
Time of diagnosis in years					
< 1 year	4	50.55(3.54)	0.72	42.08(9.05)	0.39
1 to 5 years	37	50.60(7.50)		47.81(8.75)	
> 5 years	60	49.44(7.55)		45.96(9.36)	
Gestational status					
Pregnancy	35	48.52(6.99)	0.15	46.39(8.21)	0.94
Delivery	66	50.64(7.00)		46.53(9.63)	
Infants infection status					
HIV positive/unknown	45	48.66(7.61)	0.11	46.64(8.92)	0.88
HIV negative	56	50.91(6.44)		46.35(9.36)	

### Comparison of the SF-12 scores across different dimensions of EQ-5D

Respondents indicating a health problem on the EQ-5D had significantly lower mean PCS-12 scores for all dimensions except self-care and lower mean MCS-12 scores for pain/discomfort and anxiety/depression dimensions (Table 6). The relationship between the EQ-5D functional dimensions and the PCS-12 and the relationship between the EQ-5D anxiety/depression dimension and the MCS-12 were stronger. The relationship between the less comparable dimensions and component scores was weaker.

**Table 6:** Mean (SD) SF-12 component scores by EQ-5D dimensions

EQ-5D dimension	Level	*n*	PCS-12			MCS-12		
			$\overline{\mathrm{X}}$ ± S	*P*	η-sq^a^	$\overline{\mathrm{X}}$ ± S	*P*	η-sq^a^
Mobility	1	96	50.23 ± 0.70	0.04	0.04	46.63 ± 0.94	0.48	0.005
	2	5	43.61 ± 4.02			43.68 ± 3.37		
	3	0	-			-		
Self-care	1	100	49.96 ± 0.71	0.44	0.006	46.50 ± 0.92	0.84	0.00001
	2	1	44.49 ± 0.00			45.03 ± 0.00		
	3	0	-			-		
Usual activities	1	95	50.31 ± 0.68	0.02	0.05	46.75 ± 0.89	0.25	0.01
	2	6	43.54 ± 4.14			42.28 ± 6.20		
	3	0	-			-		
Pain/discomfort	1	59	53.63 ± 5.07	0.0001	0.38	47.71 ± 8.95	0.009	0.09
	2	33	47.52 ± 7.47			46.62 ± 7.29		
	3	9	40.81 ± 6.18			37.88 ± 12.33		
Anxiety/depression	1	25	53.08 ± 3.45	0.003	0.11	54.34 ± 4.96	0.00001	0.37
	2	64	49.61 ± 6.79			45.47 ± 7.07		
	3	12	44.90 ± 10.47			35.54 ± 11.81		

^a^η-sq. = ss_model_- ss_total_ which means the strength of the of the relationship in ANOVA without the influence of sample size

### Comparison of the EQ-5D utility scores and VAS scores across different PCS-12 and MCS-12 scores

Respondents were divided according to the median SF-12 component scores. Those whose PCS-12 and MCS-12 scores were at the median or lower were classified as being in worse health, while those over the median were classified as being in better health. Significant differences were not found in the VAS scores divided by PCS-12 scores (Table 7).

**Table 7:** Comparison of those in better health (SF-12 > median) with those in worse health (SF-12 ≤ median)

SF-12	Cut off score	*n*	EQ-5D index $\overline{\mathrm{X}}$ ± S	*P*	EQ-VAS $\overline{\mathrm{X}}$ ± S	*P*
PCS-12	≤51.04	51	0.72 ± 0.12	0.0001	73.59 ± 16.23	0.14
	>51.04	50	0.82 ± 0.11		78.00 ± 13.81	
MCS-12	≤47.23	50	0.71 ± 0.08	0.0001	72.66 ± 12.49	0.04
	>47.23	51	0.83 ± 0.13		78.82 ± 13.21	

Respondents who reported no problem on each of the EQ-5D dimensions (self-reported health status was ‘11111’) were also divided according to the median SF-12 component scores. Those who scored at the median or lower were classified as being in worse health, while those who scored higher were classified as being in better health. The VAS scores were also significantly different (*P* = 0.0001) by the median split of the SF-12 scores for these subjects (Table 8).

**Table 8:** Patients reporting no problem on the EQ-5D: a comparison of those in better health (SF-12 > median) with those in worse health (SF-12 ≤ median)

SF-12	Cut off score	n	EQ-VAS $\overline{\mathrm{X}}$ ± S	*P*
PCS-12	≤54.02	9	51.95 ± 2.00	0.0001
	>54.02	8	59.29 ± 1.14	
MCS-12	≤57.27	9	53.27 ± 2.55	0.0001
	>57.17	8	58.77 ± 1.17	

### The relationship between EQ-5D and SF-12 components scores

Pearson correlation analysis showed that the EQ-5D index scores were positively correlated with both SF-12 components scores, *r* = 0.51 (*P* = 0.0001) for PCS-12 and *r* = 0.52 (*P* = 0.0001) for MCS-12, with moderate correlations. The VAS scores were also positively correlated with both SF-12 component scores, *r* = 0.24 (*P* = 0.01) for PCS-12 with a weak correlation and for MCS-12, *r* = 0.41 (*P* = 0.0001) with a moderate correlation. EQ-5D health utility scores and VAS scores were positively correlated (*r* = 0.33, *P* = 0.0001) with weak correlations (Table 9).

**Table 9:** Mutitrait-multimethod (MTMM) matrix illustrating the correlation of the EQ-5D and SF-12

	EQ-5D(utility)	EQ-5D(VAS)	SF-12(PCS)	SF-12(MCS)
EQ-5D(utility)	1			
EQ-5D(VAS)	0.33	1		
SF-12(PCS)	0.51	0.24	1	
SF-12(MCS)	0.52	0.41	0.06*	1

*P = 0.57, no statistically significant at the 0.05 level

## Discussion

Our study compared the properties of the two popularly used generic instruments, the EQ-5D and SF-12, for health-related quality of life (HRQoL) measurements in a sample of pregnant women living with HIV in Yunnan Province, China. First, we examined the discrimination ability of the SF-12 to detect the differences across degrees of EQ-5D dimensions and the discrimination ability of EQ-5D health utility scores and EQ-VAS scores to detect the differences between the cut-off scores of the SF-12. We determined both discrimination abilities of the two instruments. PCS-12 was sensitive to discriminate the differences across the levels of all EQ-5D dimensions except the self-care dimension. MCS-12 was sensitive to discriminate the differences across the levels of EQ-5D pain/discomfort and anxiety/depression dimensions. EQ-5D health utility scores and EQ-VAS scores were also sensitive enough to discriminate the differences between the cut-off scores of the SF-12. The subjects with ‘no problem’ on the EQ-5D could be divided into better and worse health groups by a median split of PCS-12 and MCS-12. SF-12 could provide a more precise description of health across the 12 dimensions. To some extent, the ability to discriminate differences between different levels of health status is an indicator to evaluate the validity of the measurements. A discrimination ability stands for a measure that could define a wide scope of potential health states [30].

Second, we assessed the correlations between EQ-5D and SF-12 component scores. The stronger relationships were observed between the EQ-5D functional dimensions and the PCS-12 and also between the EQ-5D anxiety/depression dimension and the MCS-12. Meanwhile, the correlation of the EQ-5D and SF-12 was also illustrated by a multitrait-multimethod (MTMM) matrix. EQ-5D health utility scores were moderately correlated with both SF-12 components scores. The VAS scores were also correlated with both SF-12 component scores. A MTMM matrix is generally used to evaluate construct validity of measures. In our study, construct validity was represented by a high convergent validity among the same construct measurements and low discriminate validity among the different construct measurements [31]. If we defined SF-12 as the “gold standard”, the construct validity could be identified for EQ-5D. Otherwise, the relationships between the VAS scores and both SF-12 component scores were at a low level, and a relatively stronger relationship was observed with the MCS-12, which indicated that the respective constructs of EQ-5D may not overlap [32] and that our respondents gave more weight to their mental health when they provided a total health rating.

In our study, the proportion responding ‘no problem’ on each of the EQ-5D dimensions (self-reported health status ‘11111’) was 16.8%, which was higher than the average proportion of each health status (1/243 = 0.4%). It showed that the EQ-5D dimension was not suitable to evaluate the health status of these subjects. VAS scores were less skewed and still able to distinguish the health status of the subjects who reported ‘no problem’ on the EQ-5D dimension. Therefore, EQ-VAS scores without a ceiling effect in the survey made up for the limitations of the EQ-5D dimensions, which were in accordance with other research about the specifics of EQ-5D in HRQoL measurements [33].

In our study, the expected distributions of responses, as previous studies showed [10, 11], were not observed for sociodemographic and HIV-related variables except for household income per year in the EQ-5D mobility and usual activities dimensions and education level in EQ-VAS scores. Expected relationships were also not observed for the SF-12 component scores except for PCS-12 scores in marital status. However, these results may be due to the small sample size and insufficient power to test the differences.

One limitation of this study was its small sample size. Differences may not be tested, and the rating task of a EQ-5D investigation mostly depends on a subject’s numeracy or quantitative reasoning skills. If patients had no experience in rating their health with numbers, they may perform poorly in VAS rating. Even with the limitations, our study could help to determine suitable HRQoL instruments for pregnant women living with HIV and provide evidence for the proper comparison of EQ-5D and SF-12.

## Conclusion

In conclusion, EQ-5D and SF-12 have shown discrimination abilities in measuring the HRQoL of pregnant women with HIV. The construct validity was identified for EQ-5D and SF-12 in the study. SF-12 could provide a more precise description of health across the 12 dimensions. The respective constructs of EQ-5D and EQ-VAS may not overlap. Pregnant women living with HIV in the study gave more weight to their mental health when they provided a total health rating in EQ-VAS. EQ-VAS could compensate for the limitations of the EQ-5D dimension scores with ceiling effect. The results of our study could help to determine suitable HRQoL instruments for pregnant women living with HIV and provide evidence for the proper comparison of EQ-5D and SF-12.
